# Swietenolide monohydrate

**DOI:** 10.1107/S1600536808017431

**Published:** 2008-06-13

**Authors:** Seok-Keik Tan, Hasnah Osman, Keng-Chong Wong, Hoong-Kun Fun, Suchada Chantrapromma

**Affiliations:** a School of Chemical Sciences, Universiti Sains Malaysia, 11800 USM, Penang, Malaysia; b X-ray Crystallography Unit, School of Physics, Universiti Sains Malaysia, 11800 USM, Penang, Malaysia; c Crystal Materials Research Unit, Department of Chemistry, Faculty of Science, Prince of Songkla University, Hat-Yai, Songkhla 90112, Thailand

## Abstract

The title compound, a natural b,d-*seco*-limonoid, C_27_H_34_O_8_·H_2_O, and known as Swietenolide monohydrate, has been isolated from *S. macrophylla* King. In the molecular structure, the four fused six-membered rings adopt twist-boat (ring *A*), approximate chair (ring *B*), envelope (ring *C*) and half-chair (ring *D*) conformations. The attached furan ring is essentially planar. O—H⋯O hydrogen bonds and weak C—H⋯O inter­actions connect the mol­ecules into a two-dimensional network parallel to the (100) plane. C—H⋯π inter­actions are also observed.

## Related literature

For bond-length data, see: Allen *et al.* (1987 ▶). For ring conformations, see: Cremer & Pople (1975 ▶). For related structures, see, for example: Fowles *et al.* (2007 ▶); Solomon *et al.* (2003 ▶). For the bioactivities of Swietenolide, see, for example: Chan *et al.* (1976 ▶); Jean *et al.* (2000 ▶); Kipassa *et al.* (2008 ▶); Munoz *et al.* (2000 ▶); Soediro *et al.* (1990 ▶).
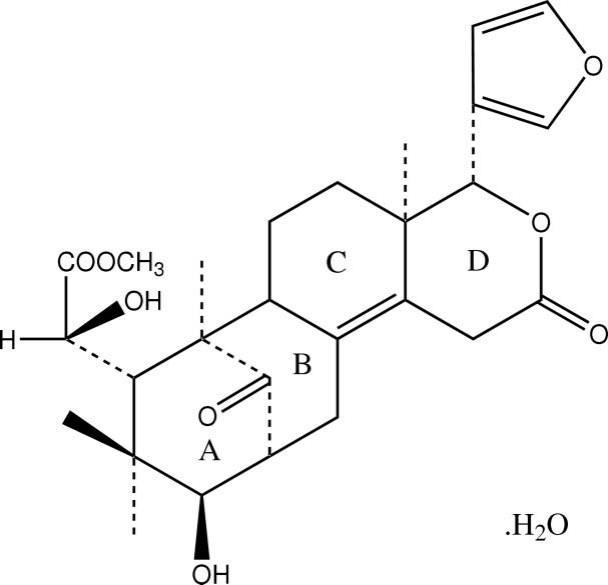


## Experimental

### 

#### Crystal data

C_27_H_34_O_8_·H_2_O
*M* *_r_* = 504.56
Monoclinic, 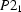

*a* = 11.5897 (1) Å
*b* = 8.8972 (1) Å
*c* = 11.7397 (1) Å
β = 90.571 (1)°
*V* = 1210.49 (2) Å^3^
*Z* = 2
Mo *K*α radiation
μ = 0.10 mm^−1^
*T* = 100.0 (1) K
0.51 × 0.26 × 0.15 mm

#### Data collection

Bruker SMART APEX2 CCD area-detector diffractometer
Absorption correction: multi-scan (**SADABS**; Bruker, 2005 ▶) *T*_min_ = 0.949, *T*_max_ = 0.985
29214 measured reflections
3748 independent reflections
3473 reflections with *I* > 2σ(*I*)
*R*_int_ = 0.035

#### Refinement

*R*[*F* ^2^ > 2σ(*F* ^2^)] = 0.036
*wR*(*F* ^2^) = 0.102
*S* = 1.06
3748 reflections
336 parameters
3 restraints
H atoms treated by a mixture of independent and constrained refinement
Δρ_max_ = 0.60 e Å^−3^
Δρ_min_ = −0.30 e Å^−3^

### 

Data collection: *APEX2* (Bruker, 2005 ▶); cell refinement: *APEX2*; data reduction: *SAINT* (Bruker, 2005 ▶); program(s) used to solve structure: *SHELXTL* (Sheldrick, 2008 ▶); program(s) used to refine structure: *SHELXTL*; molecular graphics: *SHELXTL*; software used to prepare material for publication: *SHELXTL* and *PLATON* (Spek, 2003 ▶).

## Supplementary Material

Crystal structure: contains datablocks global, I. DOI: 10.1107/S1600536808017431/is2302sup1.cif

Structure factors: contains datablocks I. DOI: 10.1107/S1600536808017431/is2302Isup2.hkl

Additional supplementary materials:  crystallographic information; 3D view; checkCIF report

## Figures and Tables

**Table 1:** Hydrogen-bond geometry (Å, °)

*D*—H⋯*A*	*D*—H	H⋯*A*	*D*⋯*A*	*D*—H⋯*A*
O2—H2*A*⋯O1*W*^i^	0.82	2.02	2.835 (2)	171
O5—H5*A*⋯O1*W*^ii^	0.82	2.05	2.760 (2)	144
O1*W*—H1*W*1⋯O1^iii^	0.84 (2)	1.98 (3)	2.809 (2)	169 (3)
O1*W*—H2*W*1⋯O6	0.842 (19)	1.994 (19)	2.821 (2)	167 (3)
C1—H1*A*⋯O1^iv^	0.98	2.38	3.325 (2)	160
C3—H3*A*⋯O2	0.98	2.57	3.032 (2)	109
C3—H3*A*⋯O7	0.98	2.40	2.861 (2)	108
C7—H7*A*⋯O2	0.97	2.34	2.690 (2)	100
C7—H7*B*⋯O4^v^	0.97	2.38	3.282 (2)	155
C21—H21*B*⋯O1	0.96	2.59	3.459 (2)	150
C21—H21*C*⋯O5	0.96	2.46	3.077 (3)	122
C27—H27*B*⋯O3	0.96	2.57	2.911 (2)	101
C23—H23*A*⋯*Cg*1^vi^	0.98	3.04	3.884 (2)	146
C25—H25*A*⋯*Cg*1^vii^	0.96	3.15	3.981 (3)	146

Symmetry codes: (i) 
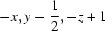
; (ii) 
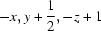
; (iii) 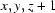; (iv) 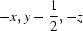; (v) 
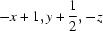
; (vi) 
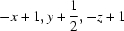
; (vii) 
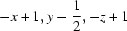
. *Cg*1 is the centroid of the C17–C20/O8 furan ring.

**Fig. 1.**
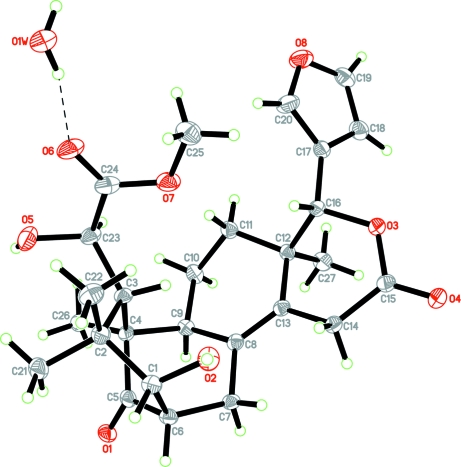
The asymmetric unit of (I), showing 50% probability displacement ellipsoids and the atomic numbering scheme. O—H···O hydrogen bond is drawn as dashed line.

**Fig. 2.**
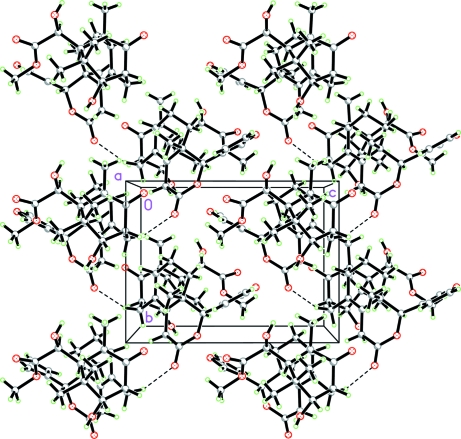
The crystal packing of the title compound viewed approximately along the a axis. Hydrogen bonds are drawn as dash lines.

## supplementary crystallographic information

### Comment

*Swietenia macrophylla* King (Meliaceae) or locally known as Big-leaf mahogany is an evergreen tree that reaches 45 to 60 meter in height. The decoction of the seeds of *Swietenia macrophylla* King was used traditionally to induce abortion, to heal wounds and to treat various skin ailments (Munoz *et al.*, 2000). In Malaysia, the seeds were ingested by local folks to provide cure for high blood pressure (Chan *et al.*, 1976). The bark extract of *Swietenia macrophylla* King was also found to be active in antimalaria activity (Soediro *et al.*, 1990). In a continual research on this plant, the leaf extracts of *S. macrophylla* were examined. The title compound, (I), (systematic name: 7,11-Methano-2*H*-cycloocta[*f*][2]benzopyran-8-acetic acid, 4-(3-furanyl)-1,4,4a,5,6,6a,7,8,9,10,11,12-dodecahydro-α,10-dihydroxy- 4a,7,9,9-tetramethyl-2,13-dioxo-methyl ester monohydrate) has been isolated from the *n*-hexane extract. It has been shown to possess biological activities such as antimalaria (Jean *et al.*, 2000) and antifeedant (Kipassa *et al.*, 2008).

The title molecule (Fig. 1) has four fused six-membered rings (*A*/*B*/*C*/*D*). The conformations adopted by rings *A*, *B*, *C* and *D* are twist boat, approximate chair, envelope and half-chair, respectively, with the puckering parameter (Cremer & Pople, 1975) Q = 0.774 (2) Å, θ = 85.0 (1)° and φ = 72.70 (15)° for ring *A*; Q = 0.642 (2) Å, θ = 161.9 (2)° and φ = 200.9 (6)° for ring *B*; Q = 0.460 (2) Å, θ = 127.4 (2)° and φ = 354.3 (3)° for ring *C*, with atom C11 displaced from the C8/C9/C10/C12/C13 plane by 0.329 (2) Å; and Q = 0.587 (2) Å, θ = 111.3 (2)° and φ = 93.72 (19)° for ring *D*, with the C12 and C16 pucker atoms deviating from the C13—C15/O3 plane by 0.343 (2) Å and -0.384 (2) Å, respectively. The furan ring (C17—C20/O8) is planar and is attached equatorially to lactone ring *D*, the torsion angle C12–C16–C17–C20 being 101.9 (2)°. The orientation of the acetic acid, 2-hydroxy-methyl ester group (C23—C25/O5—O7) at C3 can be indicated by the torsion angles of C2–C3–C23–O5 = -46.9 (2)° and C2–C3–C23–C24 = 73.2 (2)° and the methyoxyl group is slightly deviated with respect to the carbonyl group with the torsion angle C25–O7–C24–O6 of 6.5 (3)°. The bond lengths and angles in (I) are within normal ranges (Allen *et al.*, 1987) and comparable to the related structures (Fowles *et al.*, 2007; Solomon *et al.*, 2003).

In the crystal packing (Fig. 2), O—H···O hydrogen bonds and weak C—H···O interactions connect the molecules into two-dimensional network parallel to the (1 0 0) plane. O—H···O hydrogen bonds between the water and swietenolide molecules together with weak C—H···O intra- and intermolecular interactions (Table 1) play an important role in the stabilization of the crystal structure. C—H···π interactions involving furan ring (C17—C20/O8, centroid *Cg*1) are also observed in the crystal (Table 1).

### Experimental

Air-dried powdered leaves of *S. macrophylla* were extracted with *n*-hexane, CH_2_Cl_2_ and MeOH (5 *L* each) for five days respectively at room temperature. The solvents were evaporated under reduced pressure to afford *n*-hexane extract (12.8 g), CH_2_Cl_2_ extract (18.2 g) and MeOH extract (107.8 g). The *n*-hexane extract was subjected to column chromatography using silica gel with petroleum ether-ethyl acetate gradient to afford seven fractions (M1—M7). Fraction M7 (1.05 g) was further separated by preparative TLC with eluent system *n*-hexane–ethyl acetate (5:1 *v*/*v*) to afford three sub-fractions (M7a—M7c). Fraction M7b was recrystallized from CHCl_3_ to yield white single crystals of the title compound (m.p. 494–495 K).

### Refinement

Water H atoms are located in a difference map and the positional parameters were refined, with a distance restraint of O—H = 0.80 (1) Å, and with *U*_iso_(H) = 1.5*U*_eq_(O). The remaining H atoms were placed in calculated positions with d(O—H) = 0.82 Å, *U*_iso_ = 1.2*U*_eq_(O), d(C—H) = 0.97–0.98 Å, *U*_iso_ = 1.2*U*_eq_(C) for CH and aromatic, and d(C—H) = 0.96 Å, *U*_iso_ = 1.5*U*_eq_(C) for CH_3_ atoms. As there is no large anomalous dispersion for the determination of the absolute configuration, a total of 3299 Friedel pairs were merged before final refinement.

### Crystal data

**Table:**

C_27_H_34_O_8_·H_2_O	*F*_000_ = 540
*M**_r_* = 504.56	*D*_x_ = 1.384 Mg m^−^^3^
Monoclinic, *P*2_1_	Melting point = 494–495 K
Hall symbol: P 2yb	Mo *K*α radiation λ = 0.71073 Å
*a* = 11.5897 (1) Å	Cell parameters from 3748 reflections
*b* = 8.8972 (1) Å	θ = 1.7–30.0º
*c* = 11.7397 (1) Å	µ = 0.10 mm^−^^1^
β = 90.571 (1)º	*T* = 100.0 (1) K
*V* = 1210.49 (2) Å^3^	Block, white
*Z* = 2	0.51 × 0.26 × 0.15 mm

### Data collection

**Table:**

Bruker SMART APEX2 CCD area-detector diffractometer	3748 independent reflections
Radiation source: fine-focus sealed tube	3473 reflections with *I* > 2σ(*I*)
Monochromator: graphite	*R*_int_ = 0.035
Detector resolution: 8.33 pixels mm^-1^	θ_max_ = 30.0º
*T* = 100.0(1) K	θ_min_ = 1.7º
ω scans	*h* = −16→16
Absorption correction: multi-scan(*SADABS*; Bruker, 2005)	*k* = −12→12
*T*_min_ = 0.949, *T*_max_ = 0.985	*l* = −16→16
29214 measured reflections	

### Refinement

**Table:**

Refinement on *F*^2^	Secondary atom site location: difference Fourier map
Least-squares matrix: full	Hydrogen site location: inferred from neighbouring sites
*R*[*F*^2^ > 2σ(*F*^2^)] = 0.037	H atoms treated by a mixture of independent and constrained refinement
*wR*(*F*^2^) = 0.102	*w* = 1/[σ^2^(*F*_o_^2^) + (0.0598*P*)^2^ + 0.2523*P*] where *P* = (*F*_o_^2^ + 2*F*_c_^2^)/3
*S* = 1.06	(Δ/σ)_max_ = 0.001
3748 reflections	Δρ_max_ = 0.60 e Å^−^^3^
336 parameters	Δρ_min_ = −0.30 e Å^−^^3^
3 restraints	Extinction correction: none
Primary atom site location: structure-invariant direct methods	

### Special details

**Table:**

Experimental. The low-temparture data was collected with the Oxford Cyrosystem Cobra low-temperature attachment.
Geometry. All e.s.d.'s (except the e.s.d. in the dihedral angle between two l.s. planes) are estimated using the full covariance matrix. The cell e.s.d.'s are taken into account individually in the estimation of e.s.d.'s in distances, angles and torsion angles; correlations between e.s.d.'s in cell parameters are only used when they are defined by crystal symmetry. An approximate (isotropic) treatment of cell e.s.d.'s is used for estimating e.s.d.'s involving l.s. planes.
Refinement. Refinement of *F*^2^ against ALL reflections. The weighted *R*-factor *wR* and goodness of fit *S* are based on *F*^2^, conventional *R*-factors *R* are based on *F*, with *F* set to zero for negative *F*^2^. The threshold expression of *F*^2^ > σ(*F*^2^) is used only for calculating *R*-factors(gt) *etc*. and is not relevant to the choice of reflections for refinement. *R*-factors based on *F*^2^ are statistically about twice as large as those based on *F*, and *R*- factors based on ALL data will be even larger.

### Fractional atomic coordinates and isotropic or equivalent isotropic displacement parameters (Å^2^)

**Table:**

	*x*	*y*	*z*	*U*_iso_*/*U*_eq_	
O1	0.14701 (12)	0.92970 (17)	−0.07457 (11)	0.0202 (3)	
O2	0.15836 (12)	0.51412 (17)	0.17362 (12)	0.0215 (3)	
H2A	0.1147	0.4506	0.1998	0.032*	
O3	0.59393 (12)	0.49859 (16)	0.32385 (11)	0.0178 (3)	
O4	0.59920 (14)	0.30211 (18)	0.21195 (12)	0.0249 (3)	
O5	0.05304 (14)	1.03697 (19)	0.33979 (13)	0.0281 (3)	
H5A	0.0630	1.1155	0.3046	0.042*	
O6	0.09281 (15)	0.89163 (19)	0.52750 (12)	0.0286 (3)	
O7	0.23735 (14)	0.75766 (18)	0.45318 (12)	0.0254 (3)	
O8	0.63962 (14)	0.8207 (2)	0.61555 (13)	0.0307 (4)	
C1	0.09219 (16)	0.6289 (2)	0.11769 (16)	0.0174 (3)	
H1A	0.0221	0.5819	0.0869	0.021*	
C2	0.05482 (16)	0.7551 (2)	0.19977 (16)	0.0176 (3)	
C3	0.16498 (16)	0.8467 (2)	0.22950 (15)	0.0157 (3)	
H3A	0.2238	0.7719	0.2495	0.019*	
C4	0.21274 (15)	0.9309 (2)	0.12163 (15)	0.0148 (3)	
C5	0.16505 (15)	0.8578 (2)	0.01271 (16)	0.0168 (3)	
C6	0.16287 (16)	0.6893 (2)	0.01549 (16)	0.0165 (3)	
H6A	0.1268	0.6532	−0.0552	0.020*	
C7	0.29236 (15)	0.6434 (2)	0.01551 (15)	0.0160 (3)	
H7A	0.2981	0.5369	0.0322	0.019*	
H7B	0.3227	0.6592	−0.0602	0.019*	
C8	0.36654 (15)	0.7287 (2)	0.10020 (15)	0.0153 (3)	
C9	0.34658 (16)	0.8973 (2)	0.10259 (16)	0.0162 (3)	
H9A	0.3632	0.9326	0.0254	0.019*	
C10	0.43068 (17)	0.9823 (2)	0.18131 (17)	0.0199 (4)	
H10A	0.4973	1.0128	0.1376	0.024*	
H10B	0.3932	1.0726	0.2086	0.024*	
C11	0.47154 (16)	0.8905 (2)	0.28303 (16)	0.0189 (4)	
H11A	0.4065	0.8699	0.3320	0.023*	
H11B	0.5275	0.9485	0.3265	0.023*	
C12	0.52646 (15)	0.7415 (2)	0.24632 (15)	0.0150 (3)	
C13	0.44704 (16)	0.6598 (2)	0.16417 (15)	0.0152 (3)	
C14	0.46133 (16)	0.4901 (2)	0.15927 (15)	0.0171 (3)	
H14A	0.3891	0.4444	0.1820	0.021*	
H14B	0.4744	0.4619	0.0806	0.021*	
C15	0.55661 (17)	0.4233 (2)	0.23106 (16)	0.0188 (4)	
C16	0.53698 (15)	0.6395 (2)	0.35230 (15)	0.0156 (3)	
H16A	0.4589	0.6161	0.3783	0.019*	
C17	0.60331 (16)	0.7034 (2)	0.45044 (16)	0.0173 (3)	
C18	0.72511 (18)	0.7205 (3)	0.46494 (18)	0.0241 (4)	
H18A	0.7817	0.6882	0.4150	0.029*	
C19	0.74236 (19)	0.7921 (3)	0.56442 (19)	0.0246 (4)	
H19A	0.8142	0.8184	0.5942	0.030*	
C20	0.55640 (19)	0.7630 (3)	0.54466 (18)	0.0284 (5)	
H20A	0.4778	0.7646	0.5596	0.034*	
C21	−0.04291 (17)	0.8461 (3)	0.14183 (18)	0.0234 (4)	
H21A	−0.1041	0.7793	0.1193	0.035*	
H21B	−0.0134	0.8963	0.0759	0.035*	
H21C	−0.0720	0.9192	0.1944	0.035*	
C22	0.00204 (18)	0.6826 (2)	0.30642 (17)	0.0227 (4)	
H22A	−0.0592	0.6160	0.2839	0.034*	
H22B	−0.0280	0.7598	0.3550	0.034*	
H22C	0.0605	0.6270	0.3468	0.034*	
C23	0.15457 (18)	0.9491 (2)	0.33581 (15)	0.0196 (4)	
H23A	0.2207	1.0177	0.3366	0.024*	
C24	0.1553 (2)	0.8629 (2)	0.44867 (17)	0.0238 (4)	
C25	0.2369 (2)	0.6683 (3)	0.55726 (19)	0.0297 (5)	
H25A	0.2941	0.5906	0.5523	0.045*	
H25B	0.1622	0.6236	0.5667	0.045*	
H25C	0.2541	0.7318	0.6213	0.045*	
C26	0.19021 (17)	1.0997 (2)	0.11612 (16)	0.0189 (4)	
H26A	0.1087	1.1175	0.1091	0.028*	
H26B	0.2287	1.1414	0.0513	0.028*	
H26C	0.2190	1.1465	0.1844	0.028*	
C27	0.64551 (17)	0.7674 (2)	0.19245 (17)	0.0207 (4)	
H27A	0.6370	0.8298	0.1262	0.031*	
H27B	0.6782	0.6725	0.1710	0.031*	
H27C	0.6956	0.8161	0.2466	0.031*	
O1W	0.01243 (14)	0.81352 (19)	0.74563 (13)	0.0267 (3)	
H1W1	0.058 (2)	0.838 (4)	0.798 (2)	0.040*	
H2W1	0.044 (2)	0.825 (4)	0.6819 (14)	0.040*	

### Atomic displacement parameters (Å^2^)

**Table:**

	*U*^11^	*U*^22^	*U*^33^	*U*^12^	*U*^13^	*U*^23^
O1	0.0206 (6)	0.0259 (7)	0.0140 (6)	0.0037 (6)	−0.0005 (5)	0.0029 (5)
O2	0.0224 (7)	0.0211 (7)	0.0212 (7)	0.0004 (6)	0.0047 (5)	0.0050 (6)
O3	0.0198 (6)	0.0185 (6)	0.0152 (6)	0.0036 (5)	0.0005 (5)	−0.0004 (5)
O4	0.0310 (8)	0.0233 (7)	0.0205 (7)	0.0089 (6)	−0.0003 (6)	−0.0039 (6)
O5	0.0347 (9)	0.0249 (8)	0.0249 (7)	0.0099 (7)	0.0088 (6)	0.0019 (6)
O6	0.0422 (9)	0.0274 (8)	0.0161 (7)	0.0006 (7)	0.0064 (6)	−0.0004 (6)
O7	0.0362 (8)	0.0245 (7)	0.0155 (6)	0.0021 (7)	0.0004 (6)	0.0027 (6)
O8	0.0296 (8)	0.0385 (9)	0.0238 (7)	0.0001 (7)	−0.0045 (6)	−0.0118 (7)
C1	0.0154 (8)	0.0208 (9)	0.0160 (8)	−0.0025 (7)	0.0013 (6)	0.0009 (7)
C2	0.0150 (8)	0.0217 (9)	0.0162 (8)	−0.0008 (7)	0.0025 (6)	0.0003 (7)
C3	0.0187 (8)	0.0174 (8)	0.0109 (7)	0.0001 (6)	0.0019 (6)	0.0009 (6)
C4	0.0157 (8)	0.0172 (8)	0.0115 (7)	0.0016 (6)	0.0018 (6)	0.0005 (6)
C5	0.0114 (7)	0.0223 (9)	0.0167 (8)	0.0011 (7)	0.0014 (6)	0.0006 (7)
C6	0.0151 (8)	0.0214 (9)	0.0130 (8)	−0.0011 (7)	−0.0002 (6)	−0.0002 (7)
C7	0.0157 (8)	0.0194 (8)	0.0129 (7)	−0.0005 (7)	0.0014 (6)	−0.0014 (6)
C8	0.0138 (8)	0.0175 (8)	0.0145 (8)	−0.0005 (6)	0.0028 (6)	−0.0004 (6)
C9	0.0158 (8)	0.0169 (8)	0.0160 (8)	−0.0009 (6)	0.0004 (6)	0.0012 (6)
C10	0.0183 (8)	0.0161 (8)	0.0252 (9)	−0.0013 (7)	−0.0041 (7)	0.0018 (7)
C11	0.0178 (8)	0.0177 (9)	0.0211 (9)	0.0002 (7)	−0.0033 (7)	−0.0019 (7)
C12	0.0130 (7)	0.0167 (8)	0.0154 (8)	−0.0001 (6)	0.0001 (6)	0.0007 (6)
C13	0.0145 (8)	0.0173 (8)	0.0138 (8)	−0.0003 (6)	0.0029 (6)	−0.0008 (6)
C14	0.0193 (8)	0.0180 (8)	0.0141 (8)	0.0024 (7)	0.0015 (6)	−0.0005 (6)
C15	0.0212 (9)	0.0210 (9)	0.0143 (8)	0.0024 (7)	0.0027 (6)	0.0004 (7)
C16	0.0146 (8)	0.0182 (8)	0.0141 (8)	0.0011 (6)	0.0014 (6)	−0.0007 (6)
C17	0.0161 (8)	0.0187 (8)	0.0170 (8)	0.0011 (7)	−0.0004 (6)	0.0006 (7)
C18	0.0179 (9)	0.0307 (11)	0.0237 (10)	0.0016 (8)	−0.0014 (7)	0.0001 (8)
C19	0.0223 (10)	0.0262 (10)	0.0253 (10)	−0.0013 (8)	−0.0072 (8)	0.0019 (8)
C20	0.0219 (9)	0.0410 (12)	0.0223 (10)	0.0003 (9)	0.0001 (7)	−0.0116 (9)
C21	0.0162 (8)	0.0321 (11)	0.0218 (9)	0.0018 (8)	0.0020 (7)	0.0011 (8)
C22	0.0249 (10)	0.0241 (10)	0.0193 (9)	−0.0040 (8)	0.0062 (8)	0.0008 (7)
C23	0.0273 (10)	0.0183 (8)	0.0134 (8)	0.0012 (7)	0.0041 (7)	0.0004 (7)
C24	0.0360 (11)	0.0195 (9)	0.0158 (9)	−0.0037 (8)	0.0003 (7)	−0.0007 (7)
C25	0.0349 (12)	0.0294 (11)	0.0248 (10)	0.0016 (9)	−0.0009 (9)	0.0061 (9)
C26	0.0221 (9)	0.0189 (8)	0.0156 (8)	0.0026 (7)	0.0019 (7)	0.0025 (7)
C27	0.0169 (8)	0.0252 (9)	0.0200 (9)	−0.0019 (7)	0.0031 (7)	0.0035 (8)
O1W	0.0309 (8)	0.0285 (8)	0.0209 (7)	−0.0014 (7)	0.0036 (6)	−0.0020 (6)

### Geometric parameters (Å, °)

**Table:**

O1—C5	1.224 (2)	C11—C12	1.534 (3)
O2—C1	1.432 (2)	C11—H11A	0.9700
O2—H2A	0.8200	C11—H11B	0.9700
O3—C15	1.346 (2)	C12—C13	1.513 (3)
O3—C16	1.457 (2)	C12—C27	1.541 (3)
O4—C15	1.208 (2)	C12—C16	1.544 (3)
O5—C23	1.414 (2)	C13—C14	1.521 (3)
O5—H5A	0.8200	C14—C15	1.504 (3)
O6—C24	1.208 (3)	C14—H14A	0.9700
O7—C24	1.335 (3)	C14—H14B	0.9700
O7—C25	1.458 (3)	C16—C17	1.491 (3)
O8—C19	1.363 (3)	C16—H16A	0.9800
O8—C20	1.368 (3)	C17—C20	1.346 (3)
C1—C2	1.545 (3)	C17—C18	1.428 (3)
C1—C6	1.555 (3)	C18—C19	1.344 (3)
C1—H1A	0.9800	C18—H18A	0.9300
C2—C22	1.540 (3)	C19—H19A	0.9300
C2—C21	1.544 (3)	C20—H20A	0.9300
C2—C3	1.552 (3)	C21—H21A	0.9600
C3—C23	1.551 (3)	C21—H21B	0.9600
C3—C4	1.577 (2)	C21—H21C	0.9600
C3—H3A	0.9800	C22—H22A	0.9600
C4—C26	1.526 (3)	C22—H22B	0.9600
C4—C5	1.533 (3)	C22—H22C	0.9600
C4—C9	1.598 (2)	C23—C24	1.531 (3)
C5—C6	1.500 (3)	C23—H23A	0.9800
C6—C7	1.555 (3)	C25—H25A	0.9600
C6—H6A	0.9800	C25—H25B	0.9600
C7—C8	1.512 (3)	C25—H25C	0.9600
C7—H7A	0.9700	C26—H26A	0.9600
C7—H7B	0.9700	C26—H26B	0.9600
C8—C13	1.340 (3)	C26—H26C	0.9600
C8—C9	1.518 (3)	C27—H27A	0.9600
C9—C10	1.536 (3)	C27—H27B	0.9600
C9—H9A	0.9800	C27—H27C	0.9600
C10—C11	1.518 (3)	O1W—H1W1	0.834 (10)
C10—H10A	0.9700	O1W—H2W1	0.843 (10)
C10—H10B	0.9700		
			
C1—O2—H2A	109.5	C8—C13—C12	123.60 (17)
C15—O3—C16	118.08 (15)	C8—C13—C14	120.57 (17)
C23—O5—H5A	109.5	C12—C13—C14	115.82 (16)
C24—O7—C25	114.02 (17)	C15—C14—C13	116.77 (17)
C19—O8—C20	106.00 (16)	C15—C14—H14A	108.1
O2—C1—C2	112.61 (15)	C13—C14—H14A	108.1
O2—C1—C6	108.45 (15)	C15—C14—H14B	108.1
C2—C1—C6	112.51 (16)	C13—C14—H14B	108.1
O2—C1—H1A	107.7	H14A—C14—H14B	107.3
C2—C1—H1A	107.7	O4—C15—O3	117.75 (18)
C6—C1—H1A	107.7	O4—C15—C14	123.19 (18)
C22—C2—C21	106.37 (16)	O3—C15—C14	119.02 (16)
C22—C2—C1	108.60 (16)	O3—C16—C17	105.89 (15)
C21—C2—C1	108.38 (15)	O3—C16—C12	110.70 (14)
C22—C2—C3	111.68 (15)	C17—C16—C12	115.75 (16)
C21—C2—C3	114.95 (17)	O3—C16—H16A	108.1
C1—C2—C3	106.68 (14)	C17—C16—H16A	108.1
C23—C3—C2	114.79 (15)	C12—C16—H16A	108.1
C23—C3—C4	113.45 (15)	C20—C17—C18	105.48 (18)
C2—C3—C4	111.33 (14)	C20—C17—C16	125.14 (18)
C23—C3—H3A	105.4	C18—C17—C16	129.34 (17)
C2—C3—H3A	105.4	C19—C18—C17	107.01 (19)
C4—C3—H3A	105.4	C19—C18—H18A	126.5
C26—C4—C5	108.80 (15)	C17—C18—H18A	126.5
C26—C4—C3	116.15 (15)	C18—C19—O8	110.39 (19)
C5—C4—C3	109.99 (15)	C18—C19—H19A	124.8
C26—C4—C9	110.15 (15)	O8—C19—H19A	124.8
C5—C4—C9	98.41 (13)	C17—C20—O8	111.09 (19)
C3—C4—C9	111.86 (14)	C17—C20—H20A	124.5
O1—C5—C6	122.52 (18)	O8—C20—H20A	124.5
O1—C5—C4	122.26 (17)	C2—C21—H21A	109.5
C6—C5—C4	114.33 (16)	C2—C21—H21B	109.5
C5—C6—C7	104.25 (15)	H21A—C21—H21B	109.5
C5—C6—C1	111.81 (16)	C2—C21—H21C	109.5
C7—C6—C1	115.12 (16)	H21A—C21—H21C	109.5
C5—C6—H6A	108.5	H21B—C21—H21C	109.5
C7—C6—H6A	108.5	C2—C22—H22A	109.5
C1—C6—H6A	108.5	C2—C22—H22B	109.5
C8—C7—C6	114.23 (15)	H22A—C22—H22B	109.5
C8—C7—H7A	108.7	C2—C22—H22C	109.5
C6—C7—H7A	108.7	H22A—C22—H22C	109.5
C8—C7—H7B	108.7	H22B—C22—H22C	109.5
C6—C7—H7B	108.7	O5—C23—C24	104.23 (15)
H7A—C7—H7B	107.6	O5—C23—C3	115.01 (16)
C13—C8—C7	121.79 (17)	C24—C23—C3	113.71 (16)
C13—C8—C9	123.16 (17)	O5—C23—H23A	107.9
C7—C8—C9	114.99 (16)	C24—C23—H23A	107.9
C8—C9—C10	113.69 (16)	C3—C23—H23A	107.9
C8—C9—C4	109.64 (15)	O6—C24—O7	123.39 (19)
C10—C9—C4	115.77 (15)	O6—C24—C23	124.0 (2)
C8—C9—H9A	105.6	O7—C24—C23	112.54 (17)
C10—C9—H9A	105.6	O7—C25—H25A	109.5
C4—C9—H9A	105.6	O7—C25—H25B	109.5
C11—C10—C9	113.56 (16)	H25A—C25—H25B	109.5
C11—C10—H10A	108.9	O7—C25—H25C	109.5
C9—C10—H10A	108.9	H25A—C25—H25C	109.5
C11—C10—H10B	108.9	H25B—C25—H25C	109.5
C9—C10—H10B	108.9	C4—C26—H26A	109.5
H10A—C10—H10B	107.7	C4—C26—H26B	109.5
C10—C11—C12	111.75 (16)	H26A—C26—H26B	109.5
C10—C11—H11A	109.3	C4—C26—H26C	109.5
C12—C11—H11A	109.3	H26A—C26—H26C	109.5
C10—C11—H11B	109.3	H26B—C26—H26C	109.5
C12—C11—H11B	109.3	C12—C27—H27A	109.5
H11A—C11—H11B	107.9	C12—C27—H27B	109.5
C13—C12—C11	110.05 (15)	H27A—C27—H27B	109.5
C13—C12—C27	110.61 (15)	C12—C27—H27C	109.5
C11—C12—C27	111.21 (16)	H27A—C27—H27C	109.5
C13—C12—C16	105.90 (15)	H27B—C27—H27C	109.5
C11—C12—C16	108.13 (14)	H1W1—O1W—H2W1	110 (3)
C27—C12—C16	110.78 (15)		
			
O2—C1—C2—C22	−48.6 (2)	C10—C11—C12—C13	−50.7 (2)
C6—C1—C2—C22	−171.55 (16)	C10—C11—C12—C27	72.3 (2)
O2—C1—C2—C21	−163.78 (15)	C10—C11—C12—C16	−165.89 (15)
C6—C1—C2—C21	73.28 (19)	C7—C8—C13—C12	177.37 (16)
O2—C1—C2—C3	71.90 (18)	C9—C8—C13—C12	0.4 (3)
C6—C1—C2—C3	−51.0 (2)	C7—C8—C13—C14	−4.0 (3)
C22—C2—C3—C23	−44.9 (2)	C9—C8—C13—C14	178.97 (17)
C21—C2—C3—C23	76.4 (2)	C11—C12—C13—C8	23.8 (2)
C1—C2—C3—C23	−163.39 (15)	C27—C12—C13—C8	−99.5 (2)
C22—C2—C3—C4	−175.47 (16)	C16—C12—C13—C8	140.46 (17)
C21—C2—C3—C4	−54.2 (2)	C11—C12—C13—C14	−154.82 (15)
C1—C2—C3—C4	66.01 (19)	C27—C12—C13—C14	81.9 (2)
C23—C3—C4—C26	−26.9 (2)	C16—C12—C13—C14	−38.18 (19)
C2—C3—C4—C26	104.35 (19)	C8—C13—C14—C15	177.96 (16)
C23—C3—C4—C5	−151.03 (16)	C12—C13—C14—C15	−3.4 (2)
C2—C3—C4—C5	−19.7 (2)	C16—O3—C15—O4	−175.46 (16)
C23—C3—C4—C9	100.69 (18)	C16—O3—C15—C14	2.0 (2)
C2—C3—C4—C9	−128.02 (16)	C13—C14—C15—O4	−157.87 (18)
C26—C4—C5—O1	20.6 (2)	C13—C14—C15—O3	24.8 (2)
C3—C4—C5—O1	148.82 (17)	C15—O3—C16—C17	−174.57 (15)
C9—C4—C5—O1	−94.16 (19)	C15—O3—C16—C12	−48.4 (2)
C26—C4—C5—C6	−169.93 (15)	C13—C12—C16—O3	64.86 (17)
C3—C4—C5—C6	−41.7 (2)	C11—C12—C16—O3	−177.22 (14)
C9—C4—C5—C6	75.35 (17)	C27—C12—C16—O3	−55.1 (2)
O1—C5—C6—C7	101.4 (2)	C13—C12—C16—C17	−174.66 (15)
C4—C5—C6—C7	−68.06 (18)	C11—C12—C16—C17	−56.7 (2)
O1—C5—C6—C1	−133.60 (18)	C27—C12—C16—C17	65.4 (2)
C4—C5—C6—C1	56.9 (2)	O3—C16—C17—C20	−135.1 (2)
O2—C1—C6—C5	−132.57 (17)	C12—C16—C17—C20	101.9 (2)
C2—C1—C6—C5	−7.3 (2)	O3—C16—C17—C18	47.3 (3)
O2—C1—C6—C7	−13.8 (2)	C12—C16—C17—C18	−75.7 (3)
C2—C1—C6—C7	111.39 (18)	C20—C17—C18—C19	−1.6 (3)
C5—C6—C7—C8	47.4 (2)	C16—C17—C18—C19	176.3 (2)
C1—C6—C7—C8	−75.5 (2)	C17—C18—C19—O8	0.7 (3)
C6—C7—C8—C13	137.38 (17)	C20—O8—C19—C18	0.5 (3)
C6—C7—C8—C9	−45.4 (2)	C18—C17—C20—O8	2.0 (3)
C13—C8—C9—C10	2.5 (3)	C16—C17—C20—O8	−176.02 (19)
C7—C8—C9—C10	−174.69 (15)	C19—O8—C20—C17	−1.6 (3)
C13—C8—C9—C4	−128.88 (17)	C2—C3—C23—O5	−46.9 (2)
C7—C8—C9—C4	54.0 (2)	C4—C3—C23—O5	82.7 (2)
C26—C4—C9—C8	−176.16 (16)	C2—C3—C23—C24	73.2 (2)
C5—C4—C9—C8	−62.51 (17)	C4—C3—C23—C24	−157.22 (17)
C3—C4—C9—C8	53.1 (2)	C25—O7—C24—O6	6.5 (3)
C26—C4—C9—C10	53.6 (2)	C25—O7—C24—C23	−177.14 (18)
C5—C4—C9—C10	167.24 (15)	O5—C23—C24—O6	−12.4 (3)
C3—C4—C9—C10	−77.2 (2)	C3—C23—C24—O6	−138.4 (2)
C8—C9—C10—C11	−30.4 (2)	O5—C23—C24—O7	171.26 (17)
C4—C9—C10—C11	97.84 (19)	C3—C23—C24—O7	45.3 (2)
C9—C10—C11—C12	55.7 (2)		

### Hydrogen-bond geometry (Å, °)

**Table:**

*D*—H···*A*	*D*—H	H···*A*	*D*···*A*	*D*—H···*A*
O2—H2A···O1W^i^	0.82	2.02	2.835 (2)	171
O5—H5A···O1W^ii^	0.82	2.05	2.760 (2)	144
O1W—H1W1···O1^iii^	0.84 (2)	1.98 (3)	2.809 (2)	169 (3)
O1W—H2W1···O6	0.842 (19)	1.994 (19)	2.821 (2)	167 (3)
C1—H1A···O1^iv^	0.98	2.39	3.325 (2)	160
C3—H3A···O2	0.98	2.57	3.032 (2)	109
C3—H3A···O7	0.98	2.40	2.861 (2)	108
C7—H7A···O2	0.97	2.34	2.690 (2)	100
C7—H7B···O4^v^	0.97	2.38	3.282 (2)	155
C21—H21B···O1	0.96	2.59	3.459 (2)	150
C21—H21C···O5	0.96	2.46	3.077 (3)	122
C27—H27B···O3	0.96	2.57	2.911 (2)	101
C23—H23A···Cg1^vi^	0.98	3.04	3.884 (2)	146
C25—H25A···Cg1^vii^	0.96	3.15	3.981 (3)	146

Symmetry codes: (i) −*x*, *y*−1/2, −*z*+1; (ii) −*x*, *y*+1/2, −*z*+1; (iii) *x*, *y*, *z*+1;  (iv) −*x*, *y*−1/2, −*z*; (v) −*x*+1, *y*+1/2, −*z*; (vi) −*x*+1, *y*+1/2, −*z*+1; (vii) −*x*+1, *y*−1/2, −*z*+1.
